# Harnessing Genomic Analysis to Explore the Role of Telomeres in the Pathogenesis and Progression of Diabetic Kidney Disease

**DOI:** 10.3390/genes14030609

**Published:** 2023-02-28

**Authors:** Claire Hill, Seamus Duffy, Tiernan Coulter, Alexander Peter Maxwell, Amy Jayne McKnight

**Affiliations:** 1 Centre for Public Health, Queen’s University of Belfast, Belfast BT12 6BA, UK; 2 Regional Nephrology Unit, Belfast City Hospital, Belfast BT9 7AB, UK

**Keywords:** biological ageing, diabetic kidney disease, epigenetic, genetic, methylation, SNP, telomere

## Abstract

The prevalence of diabetes is increasing globally, and this trend is predicted to continue for future decades. Research is needed to uncover new ways to manage diabetes and its co-morbidities. A significant secondary complication of diabetes is kidney disease, which can ultimately result in the need for renal replacement therapy, via dialysis or transplantation. Diabetic kidney disease presents a substantial burden to patients, their families and global healthcare services. This review highlights studies that have harnessed genomic, epigenomic and functional prediction tools to uncover novel genes and pathways associated with DKD that are useful for the identification of therapeutic targets or novel biomarkers for risk stratification. Telomere length regulation is a specific pathway gaining attention recently because of its association with DKD. Researchers are employing both observational and genetics-based studies to identify telomere-related genes associated with kidney function decline in diabetes. Studies have also uncovered novel functions for telomere-related genes beyond the immediate regulation of telomere length, such as transcriptional regulation and inflammation. This review summarises studies that have revealed the potential to harness therapeutics that modulate telomere length, or the associated epigenetic modifications, for the treatment of DKD, to potentially slow renal function decline and reduce the global burden of this disease.

## 1. Introduction

The incidence of diabetes is increasing globally [1], with a 24.8% increase in the number of affected individuals expected worldwide between 2019 and 2030 (463 million increasing to 578 million) [2]. The annual global healthcare cost for diabetes is estimated to be USD 760 billion, with much of this expenditure used to treat and prevent secondary complications, such as diabetic kidney disease (DKD) [2]. DKD is associated with both type 1 diabetes (T1D) and type 2 diabetes (T2D) [3,4,5], with a higher proportion of renal dysfunction observed with T2D (42.3%) compared to T1D (32.4%) [6]. Approximately 90% of individuals with diabetes have T2D [6,7,8], highlighting the significant contribution of T2D to the prevalence of renal disease. Indeed, diabetes is the leading global cause of chronic kidney disease (CKD) and end-stage kidney disease (ESKD) [9,10,11]. 

The presence of diabetes together with CKD has been associated with higher rates of hospitalisation and increased mortality, and CKD itself is a significant independent risk factor for cardiovascular disease (CVD), an additional diabetic co-morbidity [10]. Independent risk factors for the development of CKD in diabetes have been identified, such as age, retinopathy, albuminuria, serum haemoglobin A1c levels, serum uric acid levels, and anaemia [3]. These risk factors aid the identification of those patients most at risk of disease progression, facilitating the optimisation of care and improvement in patient outcomes. DKD is a complex, multifactorial condition with environmental risk factors and inherited predisposition [12,13,14]; therefore, additional tools are required to aid earlier identification of this condition, to reduce its impact on patients and healthcare systems. 

## 2. Genomic Analysis Provides New Insights to Improve Our Understanding of Kidney Disease

Genomic analysis can provide novel insights into pathogenesis and disease progression. Genetic susceptibility to CKD has been widely explored [15], with a recent review from our group describing the impact of genetic variation, copy number variation, chromosomal rearrangement, mitochondria, sex chromosomes and telomeres on CKD susceptibility [16]. Over the past five years, a wide range of studies have explored the genomics of CKD or kidney function [17,18,19,20,21,22,23,24,25,26,27,28,29,30,31,32,33]. Multiple genomic studies have investigated kidney function decline or CKD in the context of diabetes [34,35,36,37,38,39,40,41,42]. Many of these investigations harnessed genome-wide association study (GWAS) or Mendelian Randomisation (MR) approaches, whereby genetic variants, or single nucleotide polymorphisms (SNPs), are associated with the presence of particular phenotypes. By identifying genetic variants and mapping these to particular genome regions, relevant gene, protein or molecular pathways can be identified, aiding our understanding of the molecular mechanisms potentially disrupted during disease. This knowledge can aid therapeutic development and be harnessed as a complementary tool for diagnosis and treatment planning [43]. 

In addition to genetic variation and its involvement in disease, the impact of epigenetic modifications on pathogenesis has also been explored, including in the field of DKD [12,44,45,46,47,48,49,50,51,52,53,54,55], and recently reviewed by Kato et al. [56]. Epigenetic modifications can be stably inherited or dynamically altered across the life course and can result in changes in gene expression without disrupting the genetic sequence. Individual variation with regards to exposome factors, such as diet or lifestyle, can influence renal function [57,58], highlighting the need for multi-omic studies to assess dynamic factors, such as epigenetics [12,53,54]. DNA cytosine methylation and histone modifications are recognised forms of epigenetic regulation [59,60]. DNA methylation was related to development and gene expression in the late 1970s [61,62], and is catalysed by DNA methyltransferases (DNMTs), enzymes which transfer a methyl group from S-adenyl methionine (SAM) to the fifth carbon of a cytosine nucleotide. This process forms 5 mC, predominantly in locations where a cytosine is preceded by a guanine nucleotide (a CpG site). Histone modifications were correlated with altered gene expression as early as the 1960s [63], with a range of modifications such as methylation, acetylation, phosphorylation and ubiquitination now identified [64]. 

Understanding the role of epigenetic modification on altered gene expression, as well as exploring how this translates to the development and progression of disease, can improve disease prevention and treatment. Interestingly, epigenetic modifications are reversible, making them attractive therapeutic targets [65,66]. Zhang et al. harnessed in vitro and in vivo murine tools to show that inhibition of DNA methylation mitigated kidney function decline in diabetes [67]. These authors also highlighted that the DNA methyltransferase 1 gene (*Dnmt1*) was overexpressed in diabetic mouse podocytes (epithelial cells on the outer surface of glomerular capillaries involved in kidney filtration), identifying it as a potential therapeutic target for attenuating DKD [67]. Recently, Histone Deacetylase 3 (HDAC3), involved in histone modification, has been implicated in kidney injury during diabetes, with studies identifying this as a potential therapeutic target [68,69,70].

## 3. Diabetic Kidney Disease and Ageing

**Figure 1:**
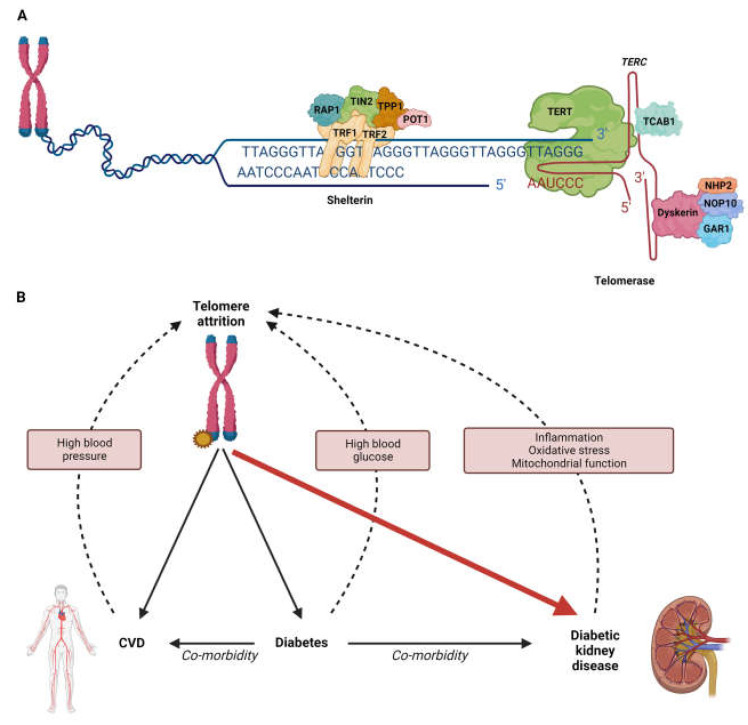
(**A**): Schematic diagram of telomeres and associated complexes. Telomeres are nucleoprotein structures at linear chromosome ends. Telomeres are made up of TTAGGG repeats, with the shelterin complex (made up of six proteins: RAP1 (Repressor/activator protein 1), POT1 (Protection of Telomeres 1), TIN2 (also known as TINF2 (TERF1-interacting nuclear factor 2)), TPP2 (Tripeptidyl peptidase 2), TRF1 (Telomeric repeat-binding factor 1) and TRF2 (Telomeric repeat-binding factor 2)) associating with this sequence. This structure protects chromosome ends from being recognised as DNA breaks. Telomerase is a ribonucleoprotein complex that maintains chromosome ends. The telomerase RNA component (TERC) acts as a template for the telomerase reverse transcription (TERT) component to generate new TTAGGG repeat DNA sequences. Accessory proteins important for telomere maintenance include TCAB1 (Telomerase Cajal body protein 1), Dyskerin, NHP2, NOP10 (Nucleolar protein 10) and GAR1 [115,116]. (**B**): Telomere shortening is associated with diabetes and common co-morbidities, cardiovascular disease (CVD) and diabetic kidney disease (DKD). Factors associated with diabetes which may contribute to telomere shortening include chronic hyperglycaemia [84] and hypertension [117]. DKD is accompanied by inflammation and oxidative stress [118], factors that have the potential to further increase the rate of telomere shortening [119,120].

DNA methylation patterns are dynamic and change with advancing age [71,72]. Indeed, DNA methylation has been identified as a genetic predictor of age [73,74,75,76]. Advancing age is a risk factor for chronic diseases such as diabetes and CKD [77,78,79,80,81], with DKD associated with accelerated kidney ageing, recently reviewed by Guo et al. [82,83,84,85]. A key component of this advanced kidney ageing is cellular senescence [84,86,87,88], whereby cells are metabolically active but no longer undergo cell division [84,89]. A key cause of cellular senescence is telomere shortening, whereby the protective nucleoprotein structures at linear chromosome ends (Figure 1A) suffer progressive loss of nucleotides during cell divisions. Telomere shortening is itself considered a marker of ageing [84,90] and has been associated with diseases common in older populations such as CVD [91,92,93,94], diabetes [77,78,79,95,96], and CKD [97,98,99,100,101]. Reduced kidney function is observed with advancing age, and some studies have correlated this phenomenon with telomere attrition [97,99,102,103,104,105,106,107,108,109,110]. The cellular senescence observed in DKD has been correlated with shorter telomere length [84], with both features observed under high extracellular glucose conditions in a cell culture model of DKD [111]. More rapid shortening of telomeres in renal cortex cells compared to the medulla has also been suggested to contribute to the glomerular senescence seen in older kidneys [102,110]. Furthermore, shorter telomeres in T2D have been associated with the presence of disease complications [80,112,113]. Akinnibosun et al. recently reviewed evidence for the association between telomeres and CKD, in both animal and human studies [114].

DKD is accompanied by inflammation and oxidative stress [118], factors with potential to further increase the rate of telomere shortening [119,120], potentially contributing to accelerated cellular senescence [84,89]. Changes in mitochondrial function, another component of biological ageing, have been associated with DKD and IgA nephropathy, as well as telomere regulation [41,49,121,122,123,124]. Additional factors associated with diabetes which may contribute to telomere shortening include chronic hyperglycaemia [84] and the upregulation of the renin–angiotensin system; individuals with hypertension have been reported to have shorter telomeres compared to those with normal blood pressure [117] (Figure 1B). Investigating the influence of telomere regulation and cellular senescence on kidney function may provide insights into how these mechanisms contribute to renal function decline.

## 4. Associations between Genetically Determined Telomere Length and Disease

GWAS and exome sequencing studies have facilitated the identification of genes involved in telomere regulation [125,126,127,128,129,130,131,132], with these genes being utilised to aid the discovery of associations between telomere length and a range of phenotypes or disease states [127,130,132,133,134,135,136,137]. These studies often utilise quantitative polymerase chain reaction (qPCR) measures of leukocyte telomere length as a proxy for telomere length in a range of other tissues. Leukocyte telomere length has been shown to positively correlate with kidney cortex telomere length [138]. Li et al. recently described their genome-wide meta-analysis, including up to 78,592 individuals of European descent, through which they identified 20 variants at 17 genomic loci significantly associated with leukocyte telomere length of genome-wide significance. Reducing their false discovery rate threshold to 0.05, they increased the number of associated variants to 52, estimated to account for approximately 2.93% of the variance observed in leukocyte telomere length [133]. Harnessing the UK Biobank as an outcome dataset for a series of MR analyses, these authors identified significant associations between shorter telomere length and conditions such as hypothyroidism, thyroid cancer, lymphoma, uterine fibroids or polyps, and benign prostatic hyperplasia [133]. Shorter telomere length was associated with a decreased risk of lung or skin cancer and leukaemia [133], consistent with previous studies [139,140,141]. 

A significant resource, useful for investigating the effects of telomere length on health and disease, was the measurement of leukocyte telomere length for 474,074 participants within the UK Biobank [142]. Harnessing these measurements, Codd et al. recently identified 138 genomic loci (108 novel) significantly associated with leukocyte telomere length, including genes with known involvement in telomere regulation, as well as genes involved in DNA replication, repair and recombination [130]. These authors assessed 93 biomedical traits and 123 diseases within the UK Biobank, determining their association with both experimentally determined and genetically determined telomere length (harnessing their significant genomic loci). Overall, genetically determined telomere length was more strongly related to most traits and diseases than experimentally determined telomere length. This study identified novel associations with circulating metabolic and endocrine biomarkers (such as insulin-like growth factor 1 (IGF-1), and lower sex hormone binding globulin), and reported novel associations between longer telomere length and increased sarcoma risk and endometriosis [130]. These authors highlighted how identifying novel telomere-related genes improves our understanding of the genomic changes that may influence telomere regulation and cellular senescence. Knowledge of how these processes become dysregulated during disease, including DKD, can improve our ability to target these pathways for diagnosis and treatment. 

## 5. Genetic Variation Influencing Telomere Regulation in Diabetic Kidney Disease

Changes in telomere length have been directly associated with DKD [96,143,144]. Telomere length correlates well with some aspects of renal function; however, it does not correlate well with all aspects, especially after adjusting for chronological age [98,99,134,139,145,146]. Table 1 summarises many studies that investigated the relationship between telomere length and measures of kidney function or disease outcome (such as CKD, DKD and diabetes). Depending on whether associations between telomere length and renal function were assessed using continuous variables (eGFR or creatinine) or discrete outcomes (presence/absence of CKD) influenced the strength of associations observed by Mazidi et al., with only continuous variables yielding significant results [99]. These authors proposed that telomere-related genes may perform additional functions, independent of their telomere maintenance roles. TERC (the RNA component of telomerase) or TERT (the telomerase reverse transcriptase) are key components of telomerase, the enzyme responsible for maintaining telomeres. These elements, however, have been shown to act as transcriptional modulators of the NF-κB pathway to promote inflammation [147,148]. Interestingly, a clinical study determined that inhibiting the NF-κB pathway to reduce inflammation slowed CKD progression [149], highlighting this as a potential therapeutic target. Additionally, Robin et al. proposed that telomeric DNA regulates genes located towards chromosome ends [150]. Considering many genes involved in DKD are located towards chromosome ends, this may be an interesting avenue for future research (Graphical abstract) [151]. 

Sun et al. presented an additional study which showed no significant difference in leukocyte telomere length between 515 healthy controls and 769 primary glomerulonephritis(GN)/CKD/ESKD patients from a Han Chinese population. These authors reported that SNPs in telomere-related genes contribute to disease susceptibility, identifying an association between the rs12696304 G allele or GG genotype (within the *TERC* gene) and GN/CKD/ESKD susceptibility in females [134]. The C allele or CC genotype frequency for rs2736100 (within the *TERT* gene) was higher in females with ESKD and not observed in females with CKD, suggesting this variant may be associated with disease progression, or may be evolutionarily selected during the disease course [134]. Genomic analysis, alongside experimentally derived telomere length measurements, can thus provide novel insights into potential mechanisms of telomere dysregulation during disease. 

Recent studies have begun to investigate genetically determined telomere length by utilising a GWAS or MR approach (Table 1) [132,135,137]. These studies identified increased risk of CKD with genetically determined telomere shortening, or due to the presence of telomere-related genetic variants. Codd et al. showed that whilst both experimentally and genetically determined leukocyte telomere length were significantly associated with many biomedical traits and diseases, CKD was only significantly associated with experimentally determined leukocyte telomere length, perhaps due to residual bias in the observational analysis or limited power [142]. Li et al. also showed in a UK Biobank cohort that genetically determined telomere attrition did not affect the risk of diseases such as diabetes or CKD [133]. These studies, together with the work described previously by Mazidi et al., highlight the importance of studying genomic and environmental features in combination, as well as investigating both continuous and discrete measures of disease, to gain a fuller understanding of disease pathogenesis and progression [12,53,54]. 

## 6. Epigenetic Variation Influencing Telomere Regulation in Diabetic Kidney Disease

As well as the genetic variation responsible for the modulation of telomere regulation during disease, epigenetic medications affecting telomere regulation have been uncovered, with this topic recently reviewed by Dogan and Forsyth [155]. Epigenetics broadly refers to the study of gene expression changes that are not the result of genetic mutation but instead due to alterations in factors such as DNA methylation, histone modifications, and non-coding RNA (ncRNA) [156]. 

Differential methylation of genes coding for telomerase has been implicated in tumorigenesis [155,157,158]. An increase in methylation within the *TERT* promotor region in cancer was associated with increased *TERT* expression, with authors proposing that this prevented the binding of the transcriptional repressor, CTCF [159,160]. However, conflicting reports exist on whether differential methylation in the *TERT* promoter results in increased or decreased expression [157,158,161,162,163]. Studies have since suggested that locus-specific methylation may be more important than overall methylation status, with Zhao et al. identifying specific epigenetic changes within the *TERT* promoter or partial exon 1 region, associated with leukaemia, which may alter the secondary or tertiary structure of the region, modifying their potential to form interactions with transcription factors and, therefore, regulating *TERT* expression [161]. Interestingly, *TERT* promoter methylation has been shown to be allele-specific, with cancer cells bearing a specific mutation in the *TERT* promoter presenting chromatin and DNA modifications different from those observed in wild-type promoters [164]. This study highlights how genetic and epigenetic changes may function together to modulate telomere regulation, emphasising the importance of assessing multi-omics to gain a full understanding of the role of telomeres in health and disease. 

Epigenetic modifications and altered gene expression can occur due to cellular stress associated with chronic diseases, including DKD [12,53,54]. Intriguingly, Tsirpanlis et al. showed decreased telomerase activity in leukocytes derived from 42 haemodialysis patients compared to 39 age-matched healthy controls, with telomerase activity significantly lower in long-term haemodialysis patients (median duration 100 months) compared to patients with a shorter duration of haemodialysis treatment (median duration 23 months) [165]. This study suggested altered telomerase activity may impact kidney function, with prior work in cancer cells suggesting that epigenetic regulation of telomerase activity may be involved in this process. However, Akinnibosun et al. summarised the mixed reports for telomerase activity levels in CKD patients, highlighting that, like telomere length itself, telomerase activity may vary across CKD stage, which should be taken into account during analyses [114].

Moreno et al. recently reviewed the influence of ncRNAs in the context of kidney disease, with specific insights included for CKD and DKD [166]. These authors highlighted ncRNAs as key molecules involved in kidney disease onset and progression, also emphasising their potential use as biomarkers or therapeutic targets [166].

Urine-derived stem cells (USCs) are stem cell-like cells which are highly proliferative and have elevated telomerase activity. Xiong et al. determined that twice as many USCs were present in DKD patient samples compared to healthy controls, with mean level of telomerase activity in USCs also significantly lower in DKD patients [167]. These authors propose telomerase activity may be an appropriate biomarker to predict DKD progression, identifying those who may have resident stem cells with reduced regenerative capacity for renal repair [167]. Interestingly, extracellular vesicles (EVs) derived from human USCs were shown to improve kidney impairment in rats with T1D, promoting angiogenesis and survival whilst inhibiting podocyte apoptosis [168]. Whilst these EVs were shown to contain cargo, such as angiogenin or growth factors, which could modulate these processes in distant cells [168], the authors did not explore telomerase protein or RNA as EV cargo. *TERT* mRNA has been identified within EVs in the context of cancer [169,170], highlighting a precedent for EV-mediated *TERT* mRNA transfer in the context of DKD. Moreover, EVs are also rich in ncRNAs [171,172], highlighting a potential additional layer of regulation whereby EV ncRNAs may modulate *TERT* mRNA action. Genomic variation associated with DKD could result in altered gene expression and disrupted telomere regulation, not only in specific cell types but non-autonomously, highlighting an interesting avenue for future research. 

## 7. Therapeutic Targeting of Telomere Regulation in Diabetic Kidney Disease

Both genetic and epigenetic variation have been implicated in the telomere dysregulation which can occur during disease. An improved understanding of this variation has proved useful in developing disease treatments and diagnostics. Research investigating telomere therapy has recently been reviewed by Hong and Yun [173] as well as Akinnibosun et al., who specifically highlighted the potential effectiveness of antidiabetic drugs to promote telomere maintenance [114,174,175]. A study potentially relevant to the development of DKD therapies was performed by Jesus et al. [176], who showed that 1 month after mice were injected with an adeno-associated virus carrying mouse *TERT* cDNA, increased *TERT* mRNA and protein levels were observed in multiple tissues, including the kidney. A subsequent increase in telomerase activity, along with a significant increase in telomere length, was observed within the kidney [176]. Fine control of gene therapy is vital to ensure that off-target effects of telomere elongation, such as triggering cancer development, do not occur. Research continues in this area; however, initial studies demonstrate reasonable safety profiles [173]. For example, Jaskelioff et al. determined that telomere integrity within their mouse model could be restored using transient expression of telomerase, without triggering carcinogenesis [177]. Beyond gene therapy, Townsley et al. have reported that treatment with Danazol, a synthetic sex hormone with androgenic properties, preserves telomere length in patients with diseases associated with telomere attrition [178], highlighting a potential use for this drug in mitigating the telomere attrition in DKD. Moreover, due to the modifiable nature of epigenetic modifications, such as DNA methylation, they are attractive therapeutic targets [12,179], including in the context of kidney disease [180,181]. Researchers have utilised the CRISPR/Cas9 system to alter promoter methylation and modify gene expression, which may be useful in therapeutic contexts [182]. However, these authors highlight that more research is needed to understand how DNA methylation correlates with the complex packaging of DNA into tertiary structures, so that improved CRISPR/Cas9 targeting can be achieved [182]. Extracellular vesicles have been explored as potential carriers of CRISPR/Cas9-based therapeutics [183], with research ongoing to optimise renal uptake of EV-loaded therapeutics [184]. EVs, together with liposomes, recombinant viruses and nanoparticles, have been highlighted as potential delivery mechanisms for RNA-based therapies to modify kidney disease-related epigenetic profiles and reduce the associated kidney damage [166].

## 8. Conclusions

With the global burden of diabetes increasing, the impact of diabetic complications, such as DKD, is growing. Improved diagnostic tools are needed to aid earlier detection of this condition and identify high-risk patients more likely to progress to renal failure. Improved diagnostic tools would provide the ability to offer early and more appropriate interventions, ultimately improving patient outcomes. Novel therapeutics are also required; however, their development depends on a better molecular understanding of DKD pathogenesis and progression. Genomic analysis can provide insights into the genes, proteins and pathways potentially dysregulated during disease. DKD is associated with advanced renal ageing and cellular senescence, with genes involved in telomere regulation identified as potential targets. Exploration of genetic and epigenetic variation of telomere regulation has identified novel telomere-related genes, with these genes predicted to not only regulate telomere length and stability, but also carry out additional functions such as transcriptional regulation and DNA repair. Novel therapies are now in development for the regulation of telomere length, with future work needed to explore their application to the prevention of cellular senescence and renal decline in DKD. 

## Figures and Tables

**Table 1:** Selection of studies investigating the association of telomere length with renal function or kidney disease, including CKD and DKD. These studies harnessed a range of methods, investigating genetically predicted telomere length, and experimentally observed telomere length.

Paper	Author, Year [Reference]	Key Findings	Method Summary	Relevance
Novel genetic determinants of telomere length from a multi-ethnic analysis of 109,122 whole genome sequences in TOPMed	Taub et al., 2022 [132]	59 novel variants associated with telomere length were identified. One SNP (rs1008438 in the HSPA1A gene) was significantly associated with risk of renal manifestations in T1D.	Whole genome sequencing (WGS) of whole blood for 109,122 individuals. TL was estimated from WGS data via the TelSeq methods. Tests for novelty were performed by checking LD with previously conducted GWAS and discarding those that had LD > 0.7 with previously described loci. PheWAS were conducted within the UK Biobank and Vanderbilt University biobank.	T1D, DKD
Polygenic basis and biomedical consequences of telomere length variation	Codd et al., 2021 [130]	Identified 193 novel variants significantly associated with leukocyte TL. No causal association between genetically estimated TL and CKD/T1D/T2D (*p* = 0.819, 0.845 and 0.163, respectively). CKD and T2D were significantly associated with experimentally determined leukocyte telomere length (*p* = 9.4527 × 10^−17^ and 0.000316, respectively).	Leukocyte TL measurements from the UK Biobank (*n* = 474,074), generated via qPCR [142]. By removing nonconditionally independent, pleiotropic and correlated variants, an instrument with 130 variants was created. MR was conducted on 93 biomedical traits and 123 disease outcomes from the UK Biobank, including CKD, T1D and T2D. For these three, the data sets contained: CKD (14,485 cases/437,060 controls); T1D (4227 cases/437,060 control); T2D (36,324 cases/437,060 control)	CKD, T1D, T2D
A Mendelian randomization study found causal linkage between telomere attrition and chronic kidney disease	Park et al., 2021 [135]	Significant causal association supporting TL shortening with increased CKD risk. IVW method (1.20 OR; 95% CI, 1.08–1.33; *p* < 0.001). All implemented MR sensitivity analyses did not affect significance. The only non-significant causal estimate was the MR-Egger regression analysis (1.10 OR; 95% CI, 0.92–1.54; *p* < 0.19) performed after SNPs with strong associations with other phenotypes (*n* = 13) were excluded. Reverse-direction MR for kidney functions effect on telomere attrition yielded significant causal estimates for all analyses excluding both the MR-Egger regressions performed. The MR-Egger intercept (*p* = 0.04) indicates the presence of directional pleiotropy in the reverse-direction MR.	A genetic instrument of 46 SNPs associated with leukocyte TL was used [133]. The SNPs were tested for genome-wide associations with confounders (hypertension, diabetes mellitus, cholesterol lowering medications, blood lipid profiles, smoking, or obesity). Summary level MR performed using European ancestry outcome data from CKDGen Consortium (*n* = 480,698, CKD cases = 41,395). Polygenic score analysis was performed using the 46 SNP instrument on UKBiobank data (Individuals with cystatin C/creatinine-eGFR data = 321,024, CKD cases = 8118). Reverse causation was investigated using a second instrument with 140 SNPs created from CKDGen GWAS data for European ancestry eGFR. This instrument was then used on UK Biobank data for individuals with TL data available (*n* = 326,075).	CKD
Association of leukocyte telomere length with chronic kidney disease in East Asians with type 2 diabetes: a Mendelian randomization study	Gurung et al., 2021 [137]	Genetically determined shorter TL was associated with increased CKD risk in patients with T2D (meta-IVW adjusted odds ratio = 1.51, 95% CI 1.12–2.12, *p* = 0.007). Similar results were obtained following sensitivity analysis. MR-Egger analysis suggested no evidence of horizontal pleiotropy.	MR analysis was performed using 16 leukocyte TL SNPs [136], investigating CKD as the outcome, defined as an eGFR of <60mL/min/1.73m^2^ (1628 cases/3140 controls). Participants were from the Singapore Study of Macro-angiopathy and Micro-vascular Reactivity in T2D and Diabetic Nephropathy cohorts.	T2D, DKD
Results from the German Chronic Kidney Disease (GCKD) study support association of relative telomere length with mortality in a large cohort of patients with moderate chronic kidney disease	Fazzini et al., 2020 [98]	RTL appeared positively associated with eGFR (*p* < 0.001) and Urine Albumin-Creatine ratio (*p* < 0.001); however this association did not remain after age and sex adjustment. Each 0.1 RTL unit decrease was associated with a 16% increase in all-cause mortality, even after age and sex adjustment. Patients in the lowest RTL quartile had a 75% higher risk for all-cause mortality than those in the highest quartile.	Relative TL was measured using qPCR within a cohort of 4955 patients from the GCKD study. Participants were divided into quartiles based on RTL and numbers of participants with confounders were presented for each quartile (smoking status, DM, prevalent CVD, sex, BMI). Average values for markers of kidney disease, BP and blood cholesterol were presented for each quartile.	CKD
The telomerase gene polymorphisms, but not telomere length, increase susceptibility to primary glomerulonephritis/end-stage kidney disease in females	Sun et al., 2020 [134]	No significant difference between TL between cases and controls. In females, a slightly shorter TL was observed in patients versus controls, but this was non-significant (*p* = 0.590). They instead identified genetic variants in telomere-related genes that contributed to disease susceptibility/progression.	515 healthy controls and 769 primary glomerulonephritis(GN)/CKD/ESKD patients from a Han Chinese population. Genomic DNA was extracted from peripheral blood. Leukocyte TL measured via qPCR. LTL was assessed in 327 controls and 592 patients.	CKD
Genome-wide Association Analysis in Humans Links Nucleotide Metabolism to Leukocyte Telomere Length	Li et al., 2020 [133]	MR analysis did not yield significant causal estimates for TL and CKD/T1D/T2D. The MR-Egger intercepts for all three indicated that directional pleiotropy was not present.	Meta-analysis of 78,592 individuals from the ENGAGE, EPIC, CVD and InterAct studies. Leukocyte TL measurements made via qPCR. A 52 SNP genetic instrument for telomere attrition was generated and used to conduct an MR investigation on 122 disease outcomes from the UK Biobank. CKD cases = 5536. T1D cases = 3469. T2D cases = 20,575.	T1D, T2D, CKD
Negative Association between Caloric Intake and Estimated Glomerular Filtration Rate in a Chinese Population: Mediation Models Involving Mitochondrial Function	Ma et al., 2020 [152]	Leukocyte TL was not significantly associated with eGFR (r = 0.056, *p* = 0.260) or urinary microalbumin to creatinine ratio (UACR) (r = 0.069, *p* = 0.168), with these associations adjusted for age.Harnessing a multiple linear regression model, these associations were also not significant (eGFR: β = 0.672 (–0.629 to 1.973), *p* = 0.310; UACR: β = 0.075 (–0.035 to 0.185), *p* = 0.183).	599 participants with different types of glucose tolerance were recruited from a Chinese rural cohort. Leukocyte TL (from peripheral blood) was determined via qPCR. Their multiple linear regression model was adjusted for age, gender, BMI, waist circumference, low-density lipoprotein cholesterol, triglycerides, abnormal glucose tolerance (including diabetes and prediabetes) and hypertension. In addition, when eGFR was a dependent variable, UACR was adjusted for; when UACR was a dependent variable, eGFR was adjusted for.	Renal function
Short Leukocyte Telomere Length Predicts Albuminuria Progression in Individuals With Type 2 Diabetes	Gurung et al., 2018 [96]	Leukocyte TL independently predicted the progression of albuminuria in T2D with preserved renal filtration function (eGFR > 60 mL/min/1.73 m^2^ and UACR < 300 mg/mg). The TL and albuminuria progression association was independent of risk factors, such as hypertension, hyperglycaemia, long diabetes duration, dyslipidaemia, and existing kidney function impairment.	A cohort of 691 Asian individuals with T2D who had preserved glomerular filtration rates. Leukocyte TL was measured via qPCR.	T2D, DKD
Peripheral blood leukocyte telomere length is associated with age but not renal function: A cross-sectional follow-up study	Zhang et al., 2018 [153]	Leukocyte TRF length was positively associated with eGFR (r = 0.182, 0.122, 0.290, and 0.254 depending on the specific eGFR calculation used, *p* < 0.01), but negatively correlated with serum cystatin C (r = −0.180, *p* < 0.01). The association with serum cystatin C was lost after adjusting for age. No association was observed between TRF length change and renal function.	Utilised a Han Chinese heathy population (*n* = 471). Telomere restriction fragment (TRF) length of genomic DNA was determined via a Southern blotting method. This study investigated Peripheral blood leukocyte telomere length. 3-year follow up TRF length data were available for 80 participants.	Renal function
Telomere attrition, kidney function, and prevalent chronic kidney disease in the United States	Mazidi et al., 2017 [99]	TL was negatively associated with urea albumin and ACR and positively associated with serum creatinine and eGFR (*p* < 0.001). In adjusted models, the association only remained significant for eGFR. Logistic regression between TL quartiles and chance of CKD did not reveal significant associations.	National Health and Nutrition Examination Survey (NHANES) cohort (*n* = 10,568). Univariable and multivariable (age, sex, race, smoking, fasting blood glucose, systolic and diastolic blood pressure, body mass index, and C-reactive protein) regression analyses were carried out. Note that diabetes and blood glucose were used as covariates. TL was measured via qPCR on whole blood-derived genomic DNA.	CKD
Association Between Telomere Length and Risk of Cancer and Non-Neoplastic Diseases: A Mendelian Randomization Study	Haycock et al., 2017 [139]	No significant association between genetically increased TL and CKD risk (0.94 OR; 95% CI, 0.77–1.16; *p* < 0.59) or T2D (1.00 OR; 95% CI, 0.84–1.20; *p* < 0.98). A statistically significant association between increased TL and lower T1D risk was reported (0.71 OR; 95% CI, 0.51–0.98; *p* < 0.04).	16 SNPs selected as genetic proxies for telomere length, derived from original GWAS reports and the NHGRI-EBI GWAS catalogue. Outcome summary data obtained for 83 diseases and 46 risk factors. CKD data were obtained from CKDGen (5807 cases/56,430 controls), with only 13 of the instrumental SNPs present in the outcome dataset. T1D dataset was obtained from T1DBase (7514 cases/9045 controls), with 13 SNPs present in the dataset. T2D data were obtained from DIAGRAM Consortium (10,415 cases/53,655 controls), with 12 SNPs present in dataset.	T1D, T2D, CKD
Association of renal function, telomere length, and markers of chronic inflammation in patients without chronic kidney and cardiovascular diseases	Pykhtina et al., 2016 [146]	Significant associations were found between TL and increased albuminuria levels (*p* = 0.023), CRP (*p* = 0.047) and fibrinogen (*p* = 0.001) even after adjustment for age and gender. No associations were found between TL and eGFR, urea levels or serum creatinine.	A cohort of 253 individuals (aged 25–85) with no chronic non-infectious diseases (cardiovascular diseases linked to atherosclerosis; arterial hypertension (AH) III degree; diabetes; CKD (glomerular filtration rate (GFR) < 60 mL/min/1.73 m^2^ or GFR ≥ 60 mL/min/1.73 m^2^ with albuminuria ≥ 30 mg/24 h), chronic and acute infectious diseases, oncological diagnoses, pregnancy, or lactation period. Measurements were performed on numerous variables (serum creatinine levels, urinary albumin level, serum fibrinogen level, blood CRP level). Note that eGFR was not measured, but derived from the MDRD equation. TL was measured via qPCR.	Renal Function
Association of relative telomere length with progression of chronic kidney disease in two cohorts: Effect modification by smoking and diabetes	Raschenberger et al., 2015 [144]	Shorter TL was a predictor of more rapid CKD progression in patients with diabetes, determining that each 0.1 unit decrease in telomere length was significantly associated with an increased hazard ratio for CKD progression of 16%.	One of the two cohorts included in this study contained patients with diabetes. A non-dialysis-dependent CKD cohort of a predominantly white population in Greater Manchester (*n* = 889). 33% of the patients had diabetes mellitus. TL measured via qPCR on whole blood-derived genomic DNA.	Diabetes (T1D and T2D), CKD, DKD
Association between kidney function and telomere length: The heart and soul study	Bansal et al., 2012 [145]	When age was included as a confounder, lower creatinine-derived eGFR, was associated with shorter telomere length at baseline (β = 9.1, 95% CI 1.2–16.9, *p* < 0.05) and predicted more rapid telomere shortening (10.8, 95% CI 4.3–17.3, *p* < 0.05) over 5 years. Once results were adjusted for age, the association was no longer statistically significant. Serum creatinine, urine creatinine clearance, cystatin C, eGFRcys, urine albumin to creatinine ratio were not significantly associated with TL.	The Heart and Soul study cohort of heart disease patients (*n* = 1024). Only 608 subjects had TL measured both at baseline and at 6 years. TL was measured via qPCR.	CKD, coronary heart disease
Telomere length and progression of diabetic nephropathy in patients with type 1 diabetes	Fyhrquist et al., 2010 [143]	TL was not significantly different between those with T1D and healthy controls, nor between healthy controls and T1D patients with normoalbuminuria (normal albumin excretion), microalbuminuria (moderate increase in albumin excretion) or macroalbuminuria (highly elevated albumin excretion). However, a higher proportion of short telomeres was an independent predictor of DKD progression (HR = 1.115, [1.039–1.195], *p* = 0.0023), alongside HbA1c and smoking.	Leukocyte TL was measured using a Southern blot technique, harnessing blood samples from 132 patients with T1D (Finnish Diabetic Nephropathy Study) and 44 healthy controls.	T1D, DKD
Telomere length predicts all-cause mortality in patients with type 1 diabetes	Astrup et al., 2010 [154]	Telomere length did not differ between patients with or without DKD. Telomere length was significantly inversely correlated to age, systolic blood pressure and duration of diabetes (*p* < 0.01).	TL was measured in 157 patients with DKD and 116 patients with persistent normoalbuminuria (Steno Diabetes Center cohort). Telomere length was measured via Southern blot from DNA samples extracted from white blood cells.	T1D, DKD

## Data Availability

No new data were created or analysed in this study. Data sharing is not applicable to this article.
